# Trauma-Induced Heterotopic Ossification Regulates the Blood-Nerve Barrier

**DOI:** 10.3389/fneur.2018.00408

**Published:** 2018-06-05

**Authors:** Zbigniew Gugala, Elizabeth A. Olmsted-Davis, Yuqing Xiong, Eleanor L. Davis, Alan R. Davis

**Affiliations:** ^1^Department of Orthopedic Surgery and Rehabilitation, University of Texas Medical Branch, Galveston, TX, United States; ^2^Center for Cell and Gene Therapy, Baylor College of Medicine, Texas Children's Hospital and Houston Methodist Hospital, Houston, TX, United States; ^3^Department of Pediatrics – Section Hematology/Oncology, Baylor College of Medicine, Houston, TX, United States; ^4^Department of Orthopedic Surgery, Baylor College of Medicine, Houston, TX, United States

**Keywords:** Blood-nerve barrier, heterotopic ossification, neuroinflammation, neural crest cells, bone matrix proteins

## Abstract

*De novo* bone formation can occur in soft tissues as a result of traumatic injury. This process, known as heterotopic ossification (HO), has recently been linked to the peripheral nervous system. Studies suggest that HO may resemble neural crest-derived bone formation and is activated through the release of key bone matrix proteins leading to opening of the blood-nerve barrier (BNB). One of the first steps in this process is the activation of a neuro-inflammatory cascade, which results in migration of chondro-osseous progenitors, and other cells from both the endoneurial and perineurial regions of the peripheral nerves. The perineurial cells undergo brown adipogenesis, to form essential support cells, which regulate expression and activation of matrix metallopeptidase 9 (MMP9) an essential regulatory protein involved in opening the BNB. However, recent studies suggest that, in mice, a key bone matrix protein, bone morphogenetic protein 2 (BMP2) is able to immediately cross the BNB to activate signaling in specific cells within the endoneurial compartment. BMP signaling correlates with bone formation and appears critical for the induction of HO. Surprisingly, several other bone matrix proteins have also been reported to regulate the BNB, leading us to question whether these matrix proteins are important in regulating the BNB. However, this temporary regulation of the BNB does not appear to result in degeneration of the peripheral nerve, but rather may represent one of the first steps in innervation of the newly forming bone.

## Overview of the BNB

**Figure 1:**
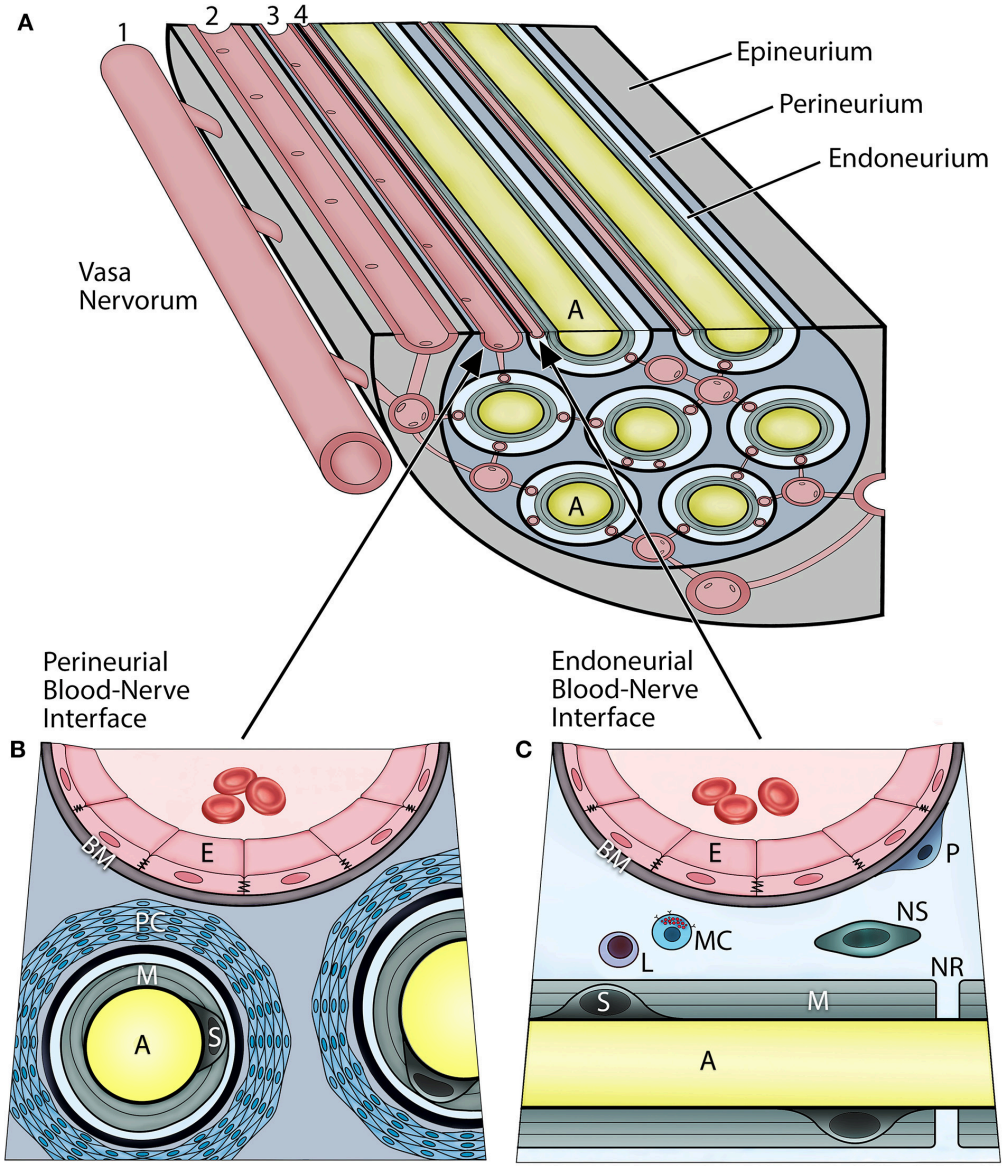
Vascular relationships within the peripheral nerve compartments and the blood-nerve barrier **(A)**. Vasa nervorum comprise an intrinsic plexus of anastomosing arterioles and capillaries that have both nutritional and functional duties for the nerve. They originate from the main artery (1) accompanying the nerve and provide radicular branches entering the nerve by perforating the epineurium. The epineurial arterioles (2) are longitudinally aligned in between nerve fascicles, and in the deeper epineurial regions they provide anastomotic connections to the perineurial capillaries (3), and subsequently endoneurial capillaries (4). The perineurial and endoneurial vessels establish a capillary network that acts in unison in maintaining a balanced hydrostatic pressure of the fluid within the endoneurium The adjacent endothelial cells of the perineurial and endoneurial capillaries are firmly connected with each other via tight junction molecules, and their basement membrane is nonfenestrated, thereby forming tight and highly regulated interfaces at which the nerve interacts with the systemic circulation. Hence, both of these perineurial and endoneurial interfaces represent structural and functional continuum—the blood-nerve barrier. Conversely, the arterioles of the epineurium exhibit open endothelial clefts and fenestrated basement membrane, therefore they are considered “leaky” to varieties of substances and tracers. The perineurium **(B)** is the sheath surrounding the axons and extends between the basement membranes of the perineurial capillaries and the endoneuria. It includes multiple concentric layers of perineurial fibroblasts combined with a dense network of collagen fibrils that surround each endoneurium. The perineurium provides the peripheral nerve structural integrity and protection against the mechanical hazards. The perineurial fibroblasts, particularly in the innermost layers, are connected with tight junctions and therefore provide a selective diffusion barrier. The perineurial blood-nerve interface is most apparent in the transperineurial vessels which connect with the endoneurial capillaries. The endoneurium (C) extends between the endoneurial capillaries and the myelin surrounding the axons. The microenvironment of the endoneurium is highly regulated, and its homeostasis maintained by both perineural and endoneurial cells. This includes the hydrostatic pressure of the endoneurial fluid, its electrolytes, albumin, nutrients, and selective cell types. The normal cellularity of the endoneurium is sparse, and consists of pericytes, mast cells, and occasional lymphocytes; whereas on the axons within layers of the myelin, the myelinating Schwann cells, and sporadically the nonmyelinating Schwann cells, are present. The endoneurium directly interfaces with the axons at the nodes of Ranvier. (A, axon; E, endothelial cell; BM, basement membrane; PC, perineurial cells; M, myelin; S, myelinating Schwann cell; P, pericyte; L, lymphocyte; MC, mast cell; NS, nonmyelinating Schwann cell; NR, node of Ranvier).

In vertebrates, the axons of peripheral nerves are structurally separated from the external environment by concentric dense connective tissue layers forming the innermost endoneurium, the inner perineurium, and the outer epineurium (Figure 1A). Such isolation not only provides mechanical protection, but also functions as a highly restrictive barrier regulating the exchange of fluids, nutrients, and cells between the nerves and their vascular supply. The BNB can be characterized as the dual interface (Figures 1B,C) comprising the capillary endothelium of the endoneurium and a multilayered cellular ensheathment of the perineurium (1–3).

The endoneurial compartment is a space surrounding myelinated or unmyelinated axons (Figure 1C). This compartment is critically important because it directly interfaces with the axons, myelin, and Schwann cells of the peripheral nerves. The endoneurial microenvironment is strictly regulated by its microvasculature. The walls of endoneurial capillaries consist of a single layer of endoneurial endothelium residing on the nonfenestrated basement membrane. These cells are at the boundary of the systemic circulation and the axonal compartment. The endoneurial endothelial cells contact each other by tight junctions which are the primary regulators of the selective BNB permeability and solute uptake. The tight junctions consist of several transmembrane proteins including zonula occludens (ZO), occludin, and claudins, specifically claudin-5. The endoneurial endothelial cells also express specific transporters, such as glucose transporter-1 (GLUT-1) and P-glycoprotein (P-gp) (4, 5). Apart from axons and Schwann cells, the endoneurial compartment also contains collagen fibrils, fluid, and other cell types. The cellular component of the endoneurial space includes the pericytes, nonmyelinating Schwann cells, and mast cells; the latter produces histamine which can further modulate permeability of the endoneurial BNB. Increased cell trafficking across the endoneurial BNB (e.g., leukocytes, macrophages) is observed in demyelinating disorders (e.g., multiple sclerosis, Guillain-Barre syndrome), neuropathic pain, and peripheral neuropathies, and is the hallmark or neurogenic inflammation (6).

The perineurial compartment extends between the perineural capillary walls and the endoneurium of the axon (Figure 1B). It contains concentric multilayered fibroblasts that surround each endoneurium. Between the perineurial fibroblasts are dense collagen type I and III bundles, which are major constituents and provide mechanical resilience to the nerve. There are numerous anastomotic connections between the perineurial and endoneurial capillaries that run obliquely across the perineurium; hence, both circulations work in unison as the BNB. Furthermore, perineurial fibroblasts are linked with each other through tight junction molecules and thereby form a selective cellular barrier, which also maintains homeostasis within the endoneurial microenvironment. In the CNS such connections are referred to as the glia limitans (7).

During mechanical injury of peripheral nerves, which may trigger HO, the nerve and its compartments undergo adaptive changes. In a crushed, stretched, or transected nerve the axons and their myelin sheath undergo degradation distal to the site of injury known as Wallerian degeneration (8). The axonal nerve body (dorsal root ganglia) activates key genes necessary for axonal regeneration. Schwann cells proliferate and create an environment conducive for axonal regeneration. Although, the permeability of the BNB increases significantly immediately after trauma (9), the perineurial cells appear resilient enough to often tolerate this change (10). There is an initial rise in perineurial permeability, which coincides with neuro-inflammation provoked by the injury as well as perineurial cell proliferation. The expression of tight junction molecules is dramatically lowered (e.g., occludins, claudins, VE-cadherins) and the leakiness of the BNB is further potentiated at the endoneurial compartment by histamine released from recruited degranulating mast cells (11). The final peak of the perineurial BNB permeability coincides with the sustained axonal regeneration, high myelinating activity of the Schwann cells, and removal of the myelin debris by macrophage, monocytes, and neutrophils during this neuroinflammatory phase (8).

## Induction of HO coincides with changes in the BNB

Recent studies to identify the molecular mechanism that leads to the onset of HO, suggest that adjacent sensory peripheral nerves play a key functional role in this process (12, 13). These studies showed that induction of HO in mouse models, resulted in *de novo* bone formation in skeletal muscle that was dependent on activation of sensory nerves, release of pain mediator's, such as substance P and CGRP, and mast cell recruitment and degranulation (14). In these studies, mice lacking the transient receptor potential cation channel subfamily V member 1 (TrpV1) resulted in significantly reduced bone formation (14). Further, induction of HO resulted in recruitment of mast cells to the nerve, whereas blocking their degranulation with cromolyn led to the suppression of bone formation (14). The essential nature of the release of pain mediators such as substance P for the induction of HO, was further demonstrated in murine studies, where an antagonist of the tachykinin receptor led to suppression of the HO (15). Recent studies by this same group confirmed the necessity for mast cell recruitment and degranulation for the induction of HO (16).

**Figure 2:**
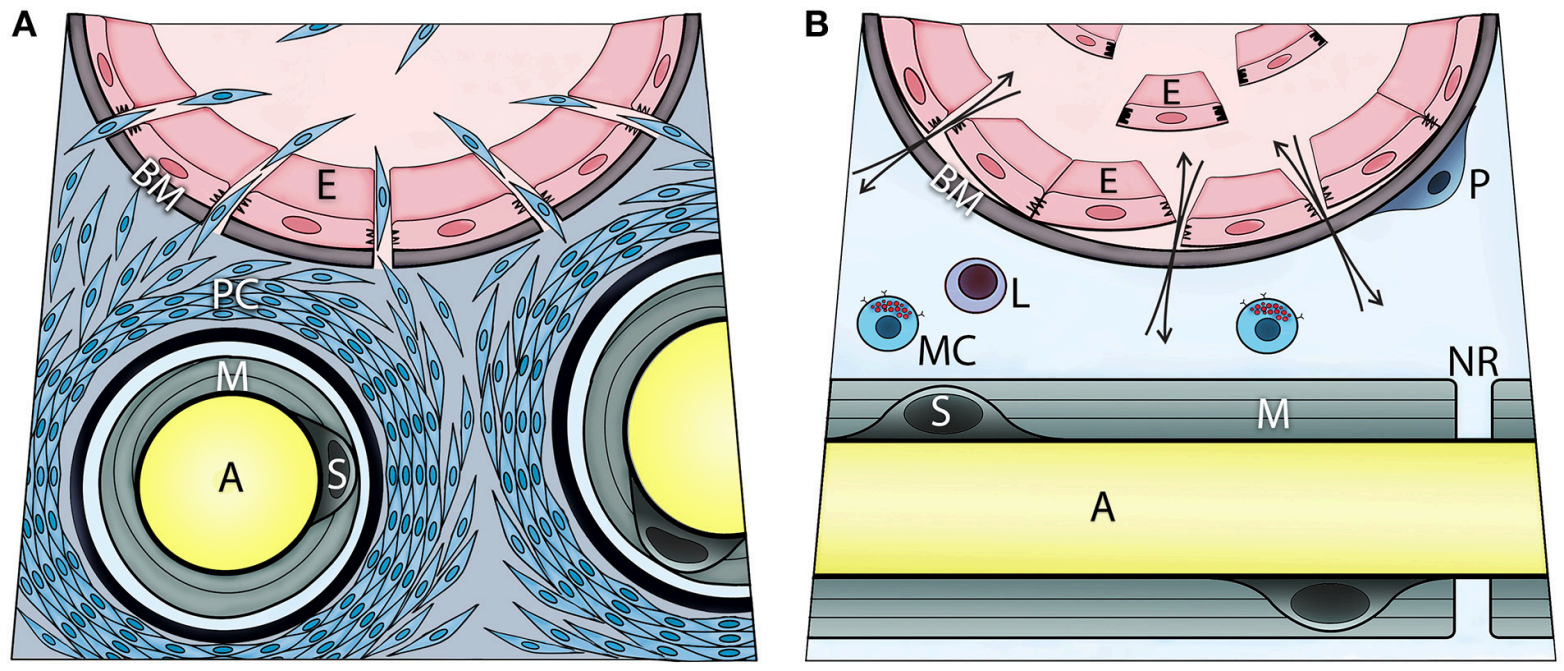
Transient opening the blood-nerve barrier upon local BMP2 delivery is associated with activation, recruitment, and migration of perineurial **(A)** and endoneurial cells **(B)**. These steps involve initial proliferation of the perineurial cells as evidenced by Ki67 immunohistochemistry, and transient expression of adrenergic receptor beta 3 (ADRB3)—a marker of brown adipogenesis. Fluorescence-activated cell sorting and analysis of cells isolated from the nerve confirmed ADRB3+ perineurial cell expansion and their expression of the neural migration marker HNK1. At 4 days post BMP2 delivery, a significant decrease in ADRB3+ cells from within the local nerves and their concurrent appearance within the adjacent soft tissue, indicate migration of the perineurial cells away from the nerve to the site of bone formation. The perineurial cells can be identified using Claudin 1 labeling. Also, cells within the endoneurium of peripheral nerves respond to BMP signaling as demonstrated by positive immunostaining for phosphorylated SMAD 1/5/8. These cells are at the endoneurial interface of the blood-nerve barrier. Upon BMP2 delivery, some of these cells are liberated to the circulation following detachment from the basement membrane and neighboring cells. These endoneurial cells uniquely express Claudin 5, particularly when liberated, and at day 7 post BMP2 delivery, were found outside their original compartment incorporating into heterotopic bone. Mast cells reside within endoneurium and their release of histamine may control the transient opening of blood-nerve barrier, and thereby cell trafficking from perineurium and endoneurium. The otherwise extremely restrictive blood-nerve barrier, when opened upon BMP2 delivery it allows also for in-and-out exchange of various solutes and thereby the neuronal contact with an external environment (A, axon; E, endothelial cell; BM, basement membrane; PC, perineurial cells; M, myelin; S, Schwann cell; P, pericyte; L, lymphocyte; MC, mast cell; NR, node of Ranvier).

Mast cell degranulation was shown to also activate the sympathetic nervous system as well as perineurial fibroblasts (17). This activation was driven by norepinephrine activation of the β3 adrenergic receptor, causing the perineurial fibroblasts to proliferate (17). Transmission electron microscopy of these nerves showed a significant increase of mitochondria in the outer layers of the perineurium and separation between the perineurial layers (data not shown). Also, these cells were observed outside the nerve and formed a continuum from the nerve to the site of bone formation. The perineurial cells express the neuro-migratory protein HNK1 (12) and potentially lead to a disruption of the BNB (Figure 2A). At the same time, brown adipogenesis was occurring (17), and the newly formed adipocytes expressed uncoupling protein 1 (UCP1), a hall mark of brown adipose tissue. Recently, we reported that these same cells were present in humans during early HO associated with traumatic injury (18).

MMP9 has previously been linked to opening of the BNB (19). In these studies, perineurial injection of hypertonic saline resulted in the binding of the noncatalytic hemepexin domain of MMP9 to low-density lipoprotein receptor 1 resulting in lowered expression of claudin 1, which is an important in the perineurial barrier. Damage of the blood nerve barrier through delivery of methamphetamine resulted in continual expression and activation of MMP9 and ILK overexpression (20). This process was inhibited by pretreatment with acetyl-L-carnitine (ALC), which inhibits BNB permeability (20). In addition, MMP9 knock-out mice appeared to be protected from ischemia because the matrix component of the barrier fails to be degraded (21). MMP9 is a gelatinase that is involved in degradation of extracellular matrix. Since the myelin sheath is mostly made of fat, MMP9 probably works non-catalytically, at least in part, by the mechanism noted above and does not degrade the myelin sheath of nerves. We (14) and others (15, 16) have shown that mast cells are an absolute requirement for HO, and inhibition of mast cell degranulation with sodium cromolyn inhibits HO (14). Although mast cells have been shown to secrete MMP9 (22), it is not known if mast cells produce MMP9 during HO.

In our murine model of HO, perineurial derived brown adipocytes were found to express high levels of MMP9 within 48 h after induction (23). Although MMP9 in the murine model was found to be the active form, surprisingly, studies using a similar model of HO in rats, revealed a lack of MMP9 activation (24). In mouse studies, MMP9 was found to be activated presumably though early neuro-inflammation (24). Conversely, the rat studies demonstrated no ectopic bone, suggesting that MMP9 activation that may be critical for HO, does not occur in the rat (24).

## Neural crest derived osteogenic stem cells

Although there are significant changes in the perineurium of peripheral nerves during HO (17), these changes occur approximately 2–4 days after the initial induction of HO. However, Lazard et al. observed significant changes in the peripheral nerves as early as 24 h after induction (25). Surprisingly, cells within the endoneurium express osteogenic transcription factors, and further characterization of these cells suggests that they also possess markers of both neural crest and osteogenic stem cells (25). These cells disappeared from the nerve within 48 h after induction of HO, and cells with similar markers could then be identified in blood and subsequently at the site of new bone formation (25). Surprisingly, the circulating cells expressed the tight junction molecule claudin 5, but were not a component of any tight junction structure (25). It was speculated that expression of claudin 5 may provide a mechanism for osteoprogenitors to traverse the BNB (Figure 2B) (25). More definitive proof that the endoneurial cells are migrating away from the nerve to form osteoblasts in HO was found from lineage tracing studies using a tamoxifen inducible Wnt1-Cre reporter mouse (26). In these studies, mice received an initial tamoxifen pulse at the time of induction of HO, which labeled the endoneurial cells. Histological analysis of the tissue 2 weeks later showed the presence of the Wnt1 reporter in osteoblasts associated with the heterotopic bone, showing that these cells had been derived from the endoneurial population (26). Tracking of chondrocytes using the Wnt-Cre reporter mouse yielded a positive result (26). Transient brown adipose tissue (tBAT) rises 70-fold on the second day after BMP2 induction, but then drops to baseline on the fourth day (17). tBAT is an energy powerhouse and uses UCP1 to create a hypoxic microenvironment for chondrogenesis (27), but also secretes angiogenic molecules (28). We have recently described its presence in humans (18). tBAT and chondrocytes appear closely related and tBAT clearly arises from the endoneurium (26), yet traverses through the perineurium (17). Whether chondrocytes take a similar path during HO remains to be determined.

## Specialized vessels carry osteoprogenitors to the site of bone formation

Osteoprogenitors cross the BNB through endoneurial vessels. However, they must then get to the site of bone formation and do this by entering the general circulation (25). Others have also reported osteoprogenitors in the general circulation (29). The vessel carrying the osteoprogenitor from the nerve to the site of bone formation is specifically structured for its task. It is small and permeable easily supporting extravasation of osteoprogenitors into the site of bone formation (25). However, like the cerebral vessels where maximum flow can rapidly be required to support neural activity, these vessels are also reinforced with vascular smooth muscle cells, pericytes, and likely other glial cells to support the vessel (30). The vessels that carry osteoprogenitors are “special” and have been identified as type H vessels that are CD31^hi^/Emcn^hi^ (31, 32).

## Other mediators of the neuroinflammatory cascade and heterotopic ossification

The neuroinflammatory cascade is essential for HO because without it, at least in species higher than the mouse, which has a very simple nerve structure, BMP2 is not able to enter the endoneurium. Even mice that lack TRPV1 have severely reduced HO (14) and blockade of the binding of substance P to its receptor blocks HO (15). Substance P induces Cox2 enabling downstream production of prostaglandins. Also, Cox2 was the number one protein induced on the second day of BMP2-induced HO (unpublished) indicating its probable importance in the neuroinflammatory process. As noted above mast cell secretion of histamine plays another important role in the neuroinflammatory cascade, and is likely that tissue-resident macrophages that are derived from yolk sac (33) play an important role as well.

There are quite a few cells that are key regulators of HO that do not participate in either bone or cartilage formation nor its turnover. Key among these is tBAT (17, 18, 27). The number of these cells increases 70-fold from Day 0 to Day 2 after BMP2 induction and then decrease to baseline after day 4 (17). These cells can control the oxygen tension in the microenvironment because they produce UCP1. Unlike the standard ATPase in the electron transport chain, UCP1 has a very high catalytic rate making it one of the few proteins that can actually burn microenvironmental oxygen (18, 27). Therefore, the pattern of hypoxia in the tissue matches exactly the pattern of tBAT (27). These cells are activated by norepinephrine through the β3 adrenergic receptor and produce HIF1, which then produces a normoxic response, but on the other side of the cell, creating adjacent hypoxic, and normoxic zones suitable for chondrogenesis and vascularization, respectively (34).

Since the heterotopic bone formed must be vascularized and innervated if it is to survive, neurites must be formed and there must be glial support cells along with vessels that guide (35) the neurovascular unit to its final destination. This probably includes Schwann cells and macrophages (35, 36), but may also include other glial cells similar to those found in the gut (37).

## Association of HO with traumatic injury to skeletal bone and the BNB

Trauma-induced HO or *de novo* bone formation is associated with injury to skeletal bone and adjacent soft tissues. Although its incidence is rare, surprisingly, an associated elevated risk has been established for particular injuries and interventions, which are associated with a higher likelihood of nerve injury. Elbow injuries have a significantly higher risk for HO than other types of fracture with the incidence reported to be from 15 to 37% [DE (38, 39)]. In another study, 30.6% of patients undergoing surgery for elbow fractures developed HO (40). In the same study, the authors noted HO was most prevalent in patients with floating elbow injury, followed by combined olecranon and radial head fractures, triad injury, and isolated radial head fractures. In addition, in their 124 patient study, the authors discovered male gender, compound fracture, fracture-dislocation, and longer time to surgery as risk factors for developing HO. Delaying surgery increased immobilization of the limb and longer immobilization was found to be correlated with HO (41). Surprisingly, in cases with significant motion or delayed treatment, bone matrix proteins may continue to be released. Further aggravation, injury, or even stretching of the ulnar nerve may result in neuro-inflammation and disruption of the BNB (42).

HO has also been linked to total hip arthroplasty (THA), or hip replacement, which is a common orthopedic procedure. Patients with a high risk in developing HO after THA include men with bilateral hypertrophic osteoarthritis, previous hip surgery, a history of HO, and patients with posttraumatic arthritis with hypertrophic osteophytosis (43–45). Patients with a moderate risk in developing HO include those with diffuse idiopathic skeletal hyperostosis, ankylosing spondylitis, or unilateral hypertrophic osteoarthritis (46–48). The occurrence of HO in patients was also discovered to be dependent on surgical techniques for THA. Certain surgical approaches to performing a THA, such as a lateral or anterolateral approach, have been shown to increase the risk of developing HO compared to a posterior approach (49, 50). In addition, the application of femoral or trochanteric osteotomy has also shown to increase risk of HO (51, 52). One of the first steps in THA is the dislocation of the damaged hip, and resection of the femoral head and neck. Depending on the technique, this could potentially lead to stretching of the adjacent sciatic nerve, which significantly disrupts the tight junction regulation and fluid homeostasis. Further, the resection of the femoral head and neck results in a significant release of bone matrix proteins within the local environment, potentially providing the essential inductive components for HO.

Limb amputation is also associated with an elevated incidence of HO (53–55). Recent U.S. combat amputees showed 36–63% with mild HO (56) and amputations conducted for blast injuries with higher injury severity scores had higher risk of developing HO (57). HO is also not limited to military combatants but can also occur in civilians affected by improvised explosive devices (IEDs). Edwards et al. (58) reported 80% of amputees, 4 out of the 5 who survived; who sustained blast-related limb injuries developed HO from the bombs in London in 2005. Of the 4 patients, all had more than 4 debridements and high injury severity score. This coincides with what Forsberg et al. (54) identified as risk factors for HO formation in military populations. However, amputation is not the only type of injury that leads to risk for HO. Traumatic tissue injury associated with bomb blasts, resulted in ~ 60–64% of patients forming HO (59). Although, in many cases the soft tissues appear uninjured, ultrasound studies detected changes suggesting damage because of the blast (60). A similar elevation in HO is also observed in civilian victims of terrorist bombings (58). The data suggests that the bomb blast temporarily disrupts the BNB leading to increased permeability and ultimately HO.

It also appears that a common cause of HO beyond injury to the peripheral nerve is the release of bone matrix proteins during or after fracture or through iatrogenic osteotomies, such as the resection of the femoral head and neck during hip replacement. We question whether certain proteins in the bone matrix are ultimately necessary for regulating the BNB after injury.

## Interaction of bone matrix proteins with the blood-nerve barrier

One of the original models of HO was developed in the 1960s by Marshall Urist, who showed that crushed skeletal bone matrix was capable of eliciting heterotopic bone formation (61). In Urist's studies (61) ground bone matrix was injected in rat muscle and bone formation occurred regardless of the location. However, in our recent studies bone formation did not occur when BMP2-producing cells were injected into rat muscle (24). This discrepancy can be explained by noting that BMP2, although an integral component of the bone matrix, is only one of many molecules, such as osteocalcin, osteonectin, and osteopontin present in bone matrix (62). Therefore, it is conceivable that even though BMP2 is necessary for bone formation, it may be insufficient at low dose. Analysis of other proteins stored in bone matrix surprisingly revealed a host of proteins known to regulate the BNB or BBB.

Recent studies in which HO was induced using BMP2, revealed that the cells undergoing BMP signaling in response to delivery of this protein were located within the endoneurium, suggesting that BNB was permeable to BMP2. Further, the level of HO correlated to both the number of endoneurial cells undergoing BMP signaling and resultant bone formation (26). Surprisingly, similar findings were observed in adjacent peripheral nerves in human tissues obtained during wound debridement that later resulted in HO (26).

Other proteins incorporated into bone matrix include Sparc, DMP1, and SPP, which have been shown to have an effect on the BBB. A secreted, acidic, cysteine-rich (SPARC, osteonectin) protein has been recently been shown to be in a class of counter-adhesive molecules (63). Studies showed that SPARC is able to break the tight junctions between endothelial cells (64), which would allow this protein to potentially open the BNB.

Small integrin-binding ligand N-linked glycoproteins (SIBLINGs) are small hydrophilic proteins that include an RGD motif that binds αvβ3 integrin. Some of the SIBLINGs activate specific metalloproteinases with integrin-binding sialoprotein (IBSP) activating MMP2; osteopontin activating MMP3, and dentin matrix acidic phosphoprotein 1 (DMP1) activating MMP9. DMP1 is produced by astrocytes in the brain, and these astrocytes exhibit tight control over the BBB. DMP1 has a glycosylation site at Ser 89. If this glycosylation site is abolished, astrocytes still produce DMP1, but the BBB is severely disrupted (65).

SPP1 or osteopontin is produced by osteoblasts and binds tightly to hydroxyapatite, which then binds osteoclasts targeting them to the bone surface. Studies of glioma have found the reactivation of an embryonic form of SPP transcriptional regulation allowing the protein to transactivate embryonic factors (66). This protein may play a key role in the transactivation of neural crest factors in the endoneurial-derived osteogenic stem cells. Further studies have shown that SPP expression is increased when the BBB is damaged, suggesting it may also play a critical role in the regulation of the BNB (67). Although these factors have not yet been shown to be essential for HO or regulatory for the BNB, their presence in the local microenvironment after traumatic injury, raises the question of their role in this process.

## Author contributions

All authors listed have made a substantial, direct and intellectual contribution to the work, and approved it for publication.

### Conflict of interest statement

The authors declare that the research was conducted in the absence of any commercial or financial relationships that could be construed as a potential conflict of interest.
